# Lifestyle in Nursing Students: Physical Activity Level, Diet Quality, Body Composition, and Cardiovascular Risk (ABSI)

**DOI:** 10.3390/healthcare13202647

**Published:** 2025-10-21

**Authors:** Carmen María Guerrero-Agenjo, Sergio Rodríguez-Cañamero, Ángel López-González, Cristina Rivera-Picón, Samantha Díaz-González, Carlos Durantez-Fernandez, Jose Alberto Laredo-Aguilera, Juan Manuel Carmona-Torres, Jesús López-Torres Hidalgo, Joseba Rabanales-Sotos

**Affiliations:** 1 Castilla-La Mancha Health Service (Servicio de Salud de Castilla-La Mancha/SESCAM), Universidad de Castilla-La Mancha/UCLM, 02071 Albacete, Spain; cmguerrero@sescam.jccm.es; 2 Group of Preventive Activities, University Health Sciences Setting (UCLM), 02071 Albacete, Spain; angel.lopez@uclm.es (Á.L.-G.); jesusl@sescam.org (J.L.-T.H.); joseba.rabanales@uclm.es (J.R.-S.); 3 Faculty of Health Sciences, Universidad de Castilla-La Mancha, Avda. Real Fábrica de Seda s/n, 45600 Talavera de la Reina, Spain; cristina.rivera@uclm.es (C.R.-P.); samanta.diaz@uclm.es (S.D.-G.); 4 Grupo de Investigación Multidisciplinar en Cuidados (IMCU), Universidad de Castilla-La Mancha, 45004 Toledo, Spain; josealberto.laredo@uclm.es (J.A.L.-A.); juanmanuel.carmona@uclm.es (J.M.C.-T.); 5 Department of Nursing, Physiotherapy and Occupatioinal Therapy, Faculty of Nursing in Albacete, Universidad de Castilla-La Mancha/UCLM, 02071 Albacete, Spain; 6 Departament of Nursing, Faculty of Nursing, University of Valladolid, 47011 Valladolid, Spain; 7 Faculty of Medicine in Albacete, Universidad de Castilla-La Mancha/UCLM, 02071 Albacete, Spain

**Keywords:** body composition, physical activity, quality of life, eating habits, university students

## Abstract

**Background/Objective**: One of the life stages that affects the consolidation of habits and health is the university stage. This transition to adulthood is associated with a decrease in physical activity, increasing the risk of cardiovascular disease. This study describes lifestyle habits related to physical activity level, diet quality, and body composition in nursing students and analyzes cardiovascular risk using the ABSI-z index. **Methods**: We conducted a cross-sectional study with 296 students from the Faculty of Nursing of Albacete (Spain). Physical activity was assessed via the IPAQ-SF. Body composition was measured by bioimpedance, from which BMI and ABSI-z scores were obtained as indicators of cardiovascular risk. The eating patterns of the participants were analyzed. **Results/Discussion**: The active students had significantly better body composition, with greater fat-free mass and muscle mass than the sedentary students, both in men (*p* = 0.037 and *p* = 0.046, respectively) and in women (*p* = 0.002 and *p* = 0.007). These findings corroborate evidence of the protective role of physical activity in maintaining metabolic health. The analysis of the ABSI-z score revealed different patterns in the distribution of body fat. High ABSI values were associated with greater abdominal girth (*p* < 0.001) and visceral fat (*p* < 0.001) in women, confirming its usefulness as an early marker of cardiovascular risk in university students. In contrast, the fulfillment of healthy dietary criteria was low, especially in the consumption of legumes (19%) and fish (25.9%). **Conclusions**: Regular physical activity is a determining factor in the body composition of university students, and ABSI is a good indicator of cardiovascular risk.

## 1. Introduction

The university stage represents a crucial moment in the life of the student due to the acquisition of new habits that will have an impact on their health [1]. During this transition to adult life, new academic responsibilities and autonomy in decision-making are acquired, with social demands being factors that can modify their patterns of nutrition, exercise, and rest, triggering a change in their body composition or quality of life [2]. Throughout this university stage, there is a decrease in the practice of physical activity (PA), accompanied by an increase in a sedentary lifestyle, which could have negative consequences for both the physical and mental health of students [3]. The relationships between the level of PA, body composition, and the perception of well-being have been studied, highlighting their influence on academic performance, emotional stability, and the prevention of chronic diseases [4].

From a physiological perspective, regular PA practice has been associated with numerous benefits, including the regulation of metabolism, maintenance of body composition, and improvement of emotional health [5]. In this context, the World Health Organization (WHO) recommends a minimum of 150 min of moderate PA or 75 min of vigorous PA weekly for adults [6]. Compliance with these recommendations contributes to improving cardiorespiratory health, reducing the probability of contracting metabolic and/or musculoskeletal diseases [6]. In contrast, a negative predisposition toward these minimum standards of the WHO causes an increase in body mass index (BMI), a greater proportion of body fat, and a reduction in physical fitness [7].

One of the criteria for assessing health status is the assessment of body composition, with which the balance between body fat and muscle tissue can be determined [8]. Maintaining a balanced distribution of these components helps prevent obesity-related diseases, such as type 2 diabetes mellitus (T2DM) and other cardiovascular disorders [9]. Different studies have shown that university students with high PA practices have a favorable relationship between muscle tissue and body fat, resulting in better metabolic efficiency and a reduction in susceptibility to diseases related to being overweight [10]. Camacho et al. (2021) reported that university students who practiced PA regularly, either at moderate or high levels, had less body fat and better functional performance than their colleagues with a sedentary lifestyle [11]. These results reinforce the importance of the practice of regular PA as a regulating factor of weight and metabolic health in university students.

In addition to physical benefits, the practice of regular PA, whose level can be evaluated with the International Physical Activity Questionnaire (IPAQ), is a fundamental component in the perception that students have about their quality of life [12,13,14]. In addition, university students who comply with PA recommendations have been shown to have a better quality of life, resulting in a lower presence of anxiety, stress, and fatigue [15]. Similarly, these benefits are not limited exclusively to improving mental health, but students also report having greater satisfaction with their perception of body image, as well as better academic performance [16].

Different factors can limit the adoption of a healthy lifestyle within the university context, despite the wide benefits recorded in the literature. Some of the barriers that hinder adherence to PA programs among university students, in addition to the exposure to and acquisition of unhealthy habits, are a lack of time, academic load, and limited access to sports facilities, or, directly, poor awareness of the need to practice PA [17].

Evidence of a positive impact on the adoption of healthy habits, with the consequent improvement in the quality of life of students, has led educational institutions to implement PA programs within academic plans and to promote the creation or remodeling of adequate spaces for sports practice and reinvent the possibility of performing PA, even in a nonperceptible way (e.g., using stairs, distancing study spaces and classrooms, etc.) [18,19].

It is necessary to analyze the relationships between PA, body composition, and quality of life in university students to develop interventions that promote a healthy lifestyle and, therefore, improve their physical and emotional well-being. Therefore, the present study proposes the following objectives for university students: to describe lifestyle habits related to physical activity level, diet quality, and body composition in nursing students and to analyze cardiovascular risk using the ABSI-z index.

## 2. Materials and Methods

### 2.1. Study Design and Sample

A descriptive, cross-sectional observational study was carried out. The study was carried out on students with a degree in nursing who were enrolled during the 2023–2024 academic year at the Faculty of Nursing of Albacete, University of Castilla–La Mancha (Spain). The inclusion criteria were as follows: students enrolled in the nursing degree of the Faculty of Nursing of Albacete and who were willing to participate in the study. The exclusion criteria were previous metabolic disease, medical contraindications to PA, pregnancy, and withdrawal from participation after being informed of the study objectives.

All the students enrolled in the first, second, and third years were invited to take part in the study, adding a total of 375 students, of whom 296 ended up participating, which represents a response rate of 78.9%.

### 2.2. Data Collection

An anonymous self-administered data collection notebook was designed for the study and was completed between February and March 2024. The following variables were recorded: sociodemographic characteristics (age, sex, type of coexistence/family nucleus, social class based on the occupation of the students’ parents according to the CSO-SEE12 [20] classification, and place of origin as appropriate for a rural or urban environment); lifestyle (toxic habits for health, such as tobacco consumption); and PA level, classifying sedentary, partially active, or active students via the International Physical Activity Questionnaire: Short Form (IPAQ-SF) [21]. Information was collected on the characteristics of their diet; the type of breakfast they ate; the frequency with which they consumed the different food groups (fruits, vegetables, dairy, meat and fish, legumes, nuts, eggs, pasta, rice and cereals, natural juices, cold cuts, sugary soft drinks, sweets, fast food, and salty snacks); and whether they followed any diet or regimen and the reason for doing so.

For the anthropometric variables, height was determined as the average of two measurements made with a Seca 222 wall height rod, and abdominal girth was the average of two measurements made with an inextensible tape measure. The evaluation of body mass was carried out via bioimpedance (SECA mBCA) with a bioimpedance meter, with which data were obtained on the amount of intracellular, extracellular, and total body water; lean mass; total fat mass; and metabolic expenditure total [22]. With these variables, the body mass index (BMI) and body shape index (ABSI) were calculated, which quantify the health risks associated with abdominal adipose tissue, taking into account sex, age, weight, height, and abdominal girth [23].

The anthropometric indicators were computed as follows: BMI was calculated using the formula BMI = weight (kg)/[height (m)]^2^. ABSI was calculated as ABSI = waist circumference (WC) (m)/[BMI^(2/3)^ × height (m)^(1/2)^]. For both indices, all measurements were expressed in International System units (kilograms and meters). BMI provides an overall estimate of relative adiposity, whereas ABSI captures central fat distribution and its association with cardiometabolic risk, offering information that complements BMI [23].

### 2.3. Ethical Considerations

This study was approved by the Committee of Ethics and Clinical Research of the Health Area of Albacete (Spain) with act number 09/2016. The research complied with the principles of the Declaration of Helsinki. To preserve the confidentiality of the participants, the data were entered into a database and identified exclusively by a numerical code both in the data collection notebook and in the computerized database (2018 Personal Data Protection Act: Ley de Protección of Personal Data and Guarantee of Digital Rights, 3/2018).

### 2.4. Data Analysis

The data obtained through the responses of the participants were processed and analyzed with the IBM SPSS Statistics v.24.0 program (IBM Corp., Armonk, NY, USA), whose license was granted by the University of Castilla–La Mancha. The characteristics of the students were analyzed via counts (n) and proportions (%) for qualitative variables and measures of central tendency (means (m) and standard deviation (SD)) for quantitative variables.

The chi-squared test was used to compare proportions between groups, with significance set at 0.05, and Student’s *t*-test was used to compare means between two groups. For comparisons with more than two groups, one-way analysis of variance (ANOVA) was performed, followed by the corresponding Tukey’s post hoc test, to assess whether there were significant differences (*p* < 0.05) between the different groups, and the data are presented as the means ± SD. The normality test used for the continuous quantitative variables was the Shapiro–Wilk test.

The IPAQ-SF is a self-administered questionnaire validated in Spain that is used to measure the frequency (days of the week), duration (time per day), and intensity (mild, moderate, or vigorous) of the PA performed during the last seven days.

The ABSI is an indicator with which an ABSI-z value is obtained that relates it to the average according to age and sex. ABSI-z classifies the results as very low risk (<−0.868), low risk (−0.868 and −0.272), average risk (−0.272 and +0.229), high risk (+0.229 and +0.798), or very high risk (>+0.798).

The quality of the diet was established according to the frequency of weekly consumption of each food group, defining the following 9 criteria for healthy eating: 3–4 servings of fish and shellfish; lean meats; eggs; 2–4 servings of legumes and dairy; 2 or more daily servings of greens and vegetables; 3 or more daily servings of fruit; 4–6 servings of cereals, bread, rice, pasta, and potatoes; and 3–7 servings of nuts per week [24]. The selection of these 9 criteria was based on the recommendations of the healthy eating pyramid for healthy adults proposed by the Spanish Society of Community Nutrition (SENC) and the Spanish Society of Family and Community Medicine (semFYC), which align with widely accepted dietary guidelines for cardiovascular and metabolic health promotion [25].

## 3. Results

The study included a total of 296 students with an average age of 20.94 years (3.17 SD) who were mostly women (83.7%). The sociodemographic characteristics and lifestyle and anthropometric measurements of the participants are shown in Table 1.

Table 2 and Table 3 show the results obtained for body composition in men according to their PA. They reported significant differences between the groups of sedentary and active men in such a way that those who had a higher level of PA had more fat-free mass. (*p* = 0.038) and greater muscle mass (*p* = 0.047) than did those who led a sedentary lifestyle. There were significant differences between active (*p* = 0.038) and partially active (*p* = 0.006) women, with more fat-free mass than sedentary women and with more muscle mass in partially active women (*p* = 0.014) than women with a more sedentary lifestyle.

The analysis of cardiovascular risk by means of the ABSI-z score is shown in Table 4, which shows significant results (*p* = 0.012) for one of the variables, total fat mass, with the main differences between the groups of men in the low-risk and high-risk groups (*p* = 0.033) and those in the very high-risk group (*p* = 0.025) (Table 4).

In women, there were significant differences in abdominal girth (*p* < 0.001) and visceral fat (*p* < 0.001). In the case of abdominal girth, these differences occurred between the following risk groups: very low compared with the average group (*p* < 0.001); very low versus high risk (*p* < 0.001); very low versus very high risk (*p* < 0.001); low versus high risk (*p* = 0.040); low versus very high risk (*p* < 0.001); and average versus very high risk (*p* = 0.041). For visceral fat, the groups that differed among themselves were very low versus high risk (*p* = 0.028); very low versus very high (*p* < 0.001); low versus very high (*p* = 0.003); and average risk versus very high risk (*p* = 0.044) (Table 5).

At a general level, when all the participants were analyzed, significant differences were detected in abdominal girth (*p* < 0.001) and visceral fat (*p* = 0.001) and, more specifically, between the very low-risk group and the average group (*p* = 0.008); the very low-risk group and the high-risk group (*p* = 0.002); the very low-risk group and the very high-risk group (*p* < 0.001); and the low-risk group and the very high-risk group (*p* < 0.001) in relation to abdominal girth and visceral fat among the very low-risk group and very high-risk group (*p* = 0.007) and the very high-risk group (*p* = 0.003). The rest of the variables analyzed, namely, BMI, relative fat mass, fat-free mass, muscle mass, weight, and height, did not significantly differ between the groups of men or women or across the entire sample (Table 6).

Table 7 shows that with respect to diet and with the data provided by the analysis, a breach of the nine criteria considered necessary for a quality diet can be observed in all the participants. Only four of them had high compliance in the majority, namely, meat (78.8%); dairy products (86.9%); fruits (70.4%); and bread, cereals, rice, pasta, and potatoes (90%). The least consumed were legumes (19%), fish and shellfish (25.9%), and eggs (27.2%). No significant differences were found between men and women in any of the food groups.

## 4. Discussion

The results obtained in this study confirm the relationship between the level of PA and body composition [24]. The findings show that university students with greater adherence to the practice of PA have a healthier body composition, characterized by lower fat mass and greater muscle mass. These results confirm those established in previous studies, in which PA was established as a regulatory agent of metabolism that helps to improve cardiorespiratory efficiency [26,27].

University students classified as active obtained significantly greater results, both men and women, in terms of fat-free mass and muscle mass than did those considered sedentary or partially active. These results corroborate what has already been established in previous research, where it was shown that the practice of regular PA helps to maintain muscle mass levels and decrease the percentage of body fat [28,29,30]. Similarly, it has already been shown that having a greater percentage of muscle mass is related to a lower risk of suffering from cardiovascular diseases, as well as metabolic diseases, highlighting the need to promote PA campaigns from an early age and their integration into plans for university teachers [31,32].

Although the benefits of the practice of PA are widely recognized, it should be noted that a large part of our population under study did not obtain them since they did not achieve the minimum PA requirements set by the WHO [33,34]. This trend has been observed in previous research, in which factors such as academic load, lack of sports facilities, or low motivation of students are related to a decrease in the practice of PA [35,36]. Moreover, the COVID-19 pandemic intensified motivational barriers via an increased mental health burden, particularly anxiety, stress, and depressive symptoms, thereby contributing to lower physical activity levels among university students [37,38]. For this reason, different authors have demonstrated the effectiveness of the incorporation of programs that promote sports practice among university students, owing to awareness campaigns, policies for access to sports facilities, and even the incorporation of physical education subjects in school curricula [16,39].

Within the analysis of sociodemographic factors, associations between PA patterns and body composition were obtained. A greater prevalence of sedentary behaviors was observed in those students who lived outside the family environment, especially in women who resided in shared flats or university residences, although this difference did not reach statistical significance (*p* = 0.688). This finding suggests that the transition toward independence during the university stage may influence the adoption of sedentary lifestyles, possibly due to changes in daily routines, the availability of sports facilities, or the loss of the family structure that previously promoted PA [40]. If we pay special attention to the group of men, a significant difference was identified according to the size of the population of origin, with a greater proportion of risk behaviors being observed in those from medium-sized localities (10,000–40,000 inhabitants; *p* = 0.004) [41]. This behavior could be explained by the differences in the availability of sports infrastructures and PA programs between different types of communities, as well as by variations in cultural norms related to physical exercise.

Regarding the analysis by sex, we obtained differentiated patterns in the PA response. Compared with men, women obtained a greater percentage of body fat, which reflects the inherent biological differences and patterns of adherence to the practice of PA documented in previous research [9,28,42]. Similarly, the general trend observed in both sexes is that greater participation in PA programs improves the results of body composition, as well as their perception of quality of life. These findings suggest that educational institutions, such as universities, should promote these PA programs as a comprehensive public health strategy.

In addition to sociodemographic and lifestyle factors, this study incorporated the ABSI-z score as a marker of cardiovascular risk, along with body composition parameters [43]. The results revealed different patterns according to sex, providing relevant information on the distribution of body fat and its relationship with metabolic risk [30]. In men, those with a very low ABSI had a significantly greater total fat mass than the groups with higher values did, which suggests that this index does not reflect the total amount of body fat but rather its distribution. However, in women, a clear association was identified between higher levels of ABSI, greater abdominal girth, and an increase in visceral fat, which indicates that a central accumulation of fat is linked to greater metabolic risk [44].

These findings are consistent with the nature of the ABSI index, which focuses on body shape and abdominal fat distribution rather than the total amount of fat mass. In men, this behavior is evidenced by the presence of greater total fat mass at low ABSI values, whereas in women, a clear relationship was observed between high ABSI values and higher values of abdominal girth and visceral fat, as has also been documented in previous studies in the general population [45]. This difference could be explained by the different patterns of fat accumulation according to sex, as has been proposed in the literature [44].

Furthermore, previous studies have shown that ABSI is a better predictor of mortality and cardiovascular risk [46] than BMI is in adult populations [47], but its use in young people has been less explored. Our data reinforce its applicability even in university students, suggesting that the ABSI can be an effective tool for the early detection of cardiovascular risk in this population. Beyond statistical associations, ABSI can be feasibly integrated into university health screening as a quick, low-cost complement to BMI to flag students with central adiposity and elevated cardiometabolic risk, even at normal BMI. Large-scale prospective studies have shown that ABSI independently predicts cardiovascular mortality [23,48,49], which supports its utility in early risk stratification. Implementation requires only simple measurements (waist circumference, height, and weight) during routine health assessments, allowing for identification of high-risk students despite normal BMI values and enabling timely interventions [23,48]. These measurement and interpretation competencies (BMI, waist circumference, and ABSI) are directly transferable to nursing curricula, strengthening training in lifestyle assessment and preventive counseling.

The results obtained show low compliance with dietary criteria among university students, among which legumes (19%) and fish/shellfish (25.9%) stand out. These findings of dietary imbalance coincide with those obtained in previous studies and are associated with increased consumption of refined carbohydrates and saturated fats, as well as foods rich in fiber and essential micronutrients. This dietary imbalance increases the risk of long-term metabolic and cardiovascular diseases [50]. The low intake of legumes and fish, together with the high consumption of refined foods and saturated fats among university students, is associated with unfavorable cardiometabolic profiles and lower micronutrient adequacy [13,51,52]. This finding is particularly concerning for health sciences students, given their future role as health prescribers, and it points to deficiencies in nutritional knowledge and practice [13]. Prior comparative studies in young adults, including sex-specific analyses, relate low adherence to healthy dietary patterns to adverse somatic indicators (BMI and waist circumference), underscoring the need to incorporate curricular and environmental strategies (e.g., guideline-based practical training and healthier campus food environments) to increase the consumption of legumes and fish and reduce refined foods [2].

This finding raises particular concern among health sciences students, considering their future responsibilities as health advocates [53]. The gap observed between theoretical understanding and personal conduct points to a pressing need for curriculum reform in nursing education. Academic programs must go beyond simply teaching about healthy lifestyles; they should embed pedagogical approaches that actively encourage students to adopt these practices themselves [54]. Research has shown that healthcare practitioners who maintain healthy habits demonstrate greater effectiveness and credibility when counseling their patients [55]. Therefore, incorporating self-care modules into nursing training could enhance the personal well-being of future nurses while simultaneously strengthening their capacity to promote community health.

Within the limitations of our study, we see how the cross-sectional design does not allow us to establish definitive causal relationships between the different variables under study and that the sample, although representative, is limited to a specific university population, which was predominantly women. In addition, factors such as perceived stress, COVID-19 pandemic-related behaviors, sleep quality and duration, and socioeconomic factors were not systematically controlled for and could influence physical activity patterns, diet quality, and body composition outcomes. In this way, the generalizability of the results could be limited with respect to other educational contexts and/or populations.

## 5. Conclusions

The present study demonstrated the relationships among the levels of PA, body composition, and cardiovascular risk of university students. Regular PA is a determining factor for maintaining healthy body composition during college and is characterized by lower fat mass and greater muscle mass, which translates into a better cardiovascular health profile.

Compared with sedentary men and women, active men and women presented significant differences in fat-free mass and muscle mass. Furthermore, the ABSI-z score proved to be a good indicator for the early detection of cardiovascular risk, especially in university-aged women, where an association between abdominal girth and a greater accumulation of visceral fat was obtained.

With respect to the fulfillment of the dietary criteria, the foods with the lowest frequency of intake were legumes, along with fish/shellfish. However, the foods with the highest frequency of intake were meats, dairy products, bread, cereals, rice, pasta, and potatoes. This dietary imbalance, associated with increased consumption of refined carbohydrates and saturated fats, increases the risk of cardiovascular diseases in the long term.

For this reason, we believe that carrying out future longitudinal studies that evaluate changes in physical activity and dietary habits throughout nursing education would make it possible to identify critical moments for intervention and to evaluate the effectiveness of the educational strategies designed to promote healthy lifestyles within this population.

## Figures and Tables

**Table 1:** Sociodemographic, anthropometric, and body composition characteristics of the study subjects.

	Menu (*n* = 48)	Women (*n* = 248)	Total (*n* = 296)
Age (years)			
18–19	16 (33.4)	67 (27.0)	83 (28.0)
20–21	23 (47.9)	119 (47.9)	142 (48.0)
22–23	4 (8.4)	40 (16.1)	44 (14.9)
24 or over	5 (10.3)	22 (9.0)	27 (9.1)
Academic year			
First	22 (45.8)	78 (31.5)	100 (33.8)
Second	13 (27.1)	87 (35.1)	100 (33.8)
Third	13 (27.1)	83 (33.5)	96 (32.4)
Population of origin (inhabitants)			
<10,000	9 (19.1)	67 (27.4)	76 (26.2)
10,000–40,000	2 (4.3)	38 (15.6)	40 (13.7)
>40,000	36 (76.6)	139 (57.0)	175 (60.1)
Living situation			
Living alone	0 (0.0)	2 (0.8)	2 (0.7)
Living with partner or married, with/without children	1 (2.1)	7 (2.8)	8 (2.7)
Living with parents, siblings, etc.	43 (91.5)	162 (65.6)	205 (69.7)
Living in shared accommodation	1 (2.1)	56 (22.7)	57 (19.4)
Living in a residence	2 (4.3)	20 (8.1)	22 (7.5)
Smoker			
Yes	6 (12.8)	36 (14.5)	42 (14.2)
No	41 (87.2)	212 (85.5)	253 (85.8)
Physical exercise			
Sedentary	8 (16.7)	29 (11.8)	37 (12.6)
Partially active	18 (37.5)	107 (43.5)	125 (42.5)
Active	22 (45.8)	110 (44.7)	132 (44.9)
Social class *			
I	7 (15.2)	19 (8.1)	26 (9.3)
II	8 (17.4)	41 (17.4)	49 (17.4)
III (a, b)	8 (17.4)	44 (18.7)	52 (18.5)
IV (a, b, c)–VI	3 (6.5)	16 (6.8)	19 (6.8)
VII (a, b)	20 (43.5)	115 (48.9)	135 (48.0)
ABSI-z score			
Very low	8 (16.7)	47 (19.1)	55 (18.7)
Low	11 (22.9)	39 (15.9)	50 (17.0)
Average	9 (18.8)	62 (25.2)	71 (24.1)
High	15 (31.1)	40 (16.3)	55 (18.7)
Very high	5 (10.4)	58 (23.6)	63 (21.4)
BMI	22.63 ± 3.07	22.22 ± 3.49	22.29 ± 3.42
Relative fat mass	21.73 ± 7.33	27.44 ± 7.35	26.51 ± 7.63
Total fat mass	14.32 ± 5.52	16.89 ± 7.00	16.48 ± 6.84
Fat free mass	51.72 ± 10.87	43.29 ± 7.04	44.66 ± 8.37
Muscle mass	24.31 ± 6.29	19.35 ± 4.11	20.16 ± 4.88
Abdominal girth	79.31± 9.22	77.37 ± 9.72	77.69 ± 9.65
Weight	65.91 ± 12.64	60.22 ± 11.07	61.14 ± 11.51
Height	170.16 ± 9.09	164.43 ± 7.13	165.36 ± 7.76
Visceral fat	0.82 ± 0.67	0.72 ± 1.03	0.73 ± 0.98

* I: Higher-grade professionals, administrators, and officials; managers in large industrial establishments; large proprietors. II: Lower-grade professionals, administrators, and officials, higher-grade technicians; managers in small industrial establishments; supervisors of nonmanual employees. IIIa: Routine nonmanual employees, higher grade (administration and commerce). IIIb: Routine nonmanual employees, lower grade (sales and services). IVa: Small proprietors, artisans, etc., with employees. IVb: Small proprietors, artisans, etc., with employees. IVc: Farmers and smallholders; other self-employed workers in primary production. V: Lower-grade technicians; supervisors of manual workers. VI: Skilled manual workers. VIIa: Semiskilled and unskilled manual workers (not in agriculture, etc.). VIIb: Agricultural and other workers in primary production.

**Table 2:** Body composition of men according to level of physical activity.

	Sedentary	Partially Active	Active	*p*-Value
BMI	21.10 ± 1.26	22.62 ± 3.19	23.20 ± 3.33	0.258
Relative fat mass	24.03 ± 6.65	20.73 ± 7.32	21.72 ± 7.69	0.582
Total fat mass	13.33 ± 3.24	14.00 ± 5.88	14.95 ± 5.99	0.748
Fat free mass	42.91 ± 6.72 ^a^	52.87 ± 9.85 ^ab^	53.99 ± 11.60 ^b^	0.037 *
Muscle mass	19.39 ± 3.82 ^a^	24.91 ± 5.67 ^ab^	25.61 ± 6.80 ^b^	0.046 *
Abdominal girth	73.00 ± 4.59	80.27 ± 9.09	80.81 ± 9.90	0.102
Weight	56.24 ± 4.21	66.88 ±12.26	68.62 ± 13.64	0.052
Height	163.25 ±4.52	171.61 ± 9.64	171.50 ± 9.09	0.059
Visceral fat	0.46 ± 0.13	0.87 ± 0.81	0.91 ± 0.64	0.250

One-way analysis of variance (ANOVA) was performed, followed by Tukey’s post hoc test for multiple comparisons, and the data are presented as mean ± SD, n = 48 men. The classification of the level of physical activity was carried out via the International Physical Activity Questionnaire (IPAQ), categorizing the participants as sedentary, partially active, or active. The means in a row without a common superscript letter differ significantly (*p* < 0.05 *).

**Table 3:** Body composition of women according to level of physical activity.

	Sedentary	Partially Active	Active	*p*-Value
BMI	22.46 ± 4.75	21.99 ± 3.87	22.36 ± 2.66	0.686
Relative fat mass	29.12 ± 7.72	28.10 ± 7.47	26.31 ± 7.08	0.084
Total fat mass	18.13 ± 8.63	17.23 ± 7.87	16.22 ± 5.54	0.344
Fat free mass	41.37 ± 3.71 ^a^	42.06 ± 7.14 ^a^	45.00 ± 7.32 ^b^	0.002 *
Muscle mass	18.35 ± 2.45 ^ab^	18.69 ± 4.32 ^a^	20.27 ± 4.22 ^b^	0.007 *
Abdominal girth	77.27 ± 12.12	77.32 ± 10.96	77.37 ± 7.65	0.999
Weight:	59.51 ± 11.79	59.36 ± 12.98	61.23 ± 8.72	0.436
Height	163.06 ± 5.82	163.91 ± 7.77	165.34 ± 6.75	0.182
Visceral fat	0.66 ± 0.64	0.77 ± 0.77	0.68 ± 1.32	0.759

One-way analysis of variance (ANOVA) was performed, followed by Tukey’s post hoc test for multiple comparisons, and the data are presented as mean ± SD, n = 248 women. The classification of the level of physical activity was carried out via the International Physical Activity Questionnaire (IPAQ), categorizing the participants as sedentary, partially active, or active. The means in a row without a common superscript letter differ significantly (*p* < 0.05 *).

**Table 4:** Body composition of men according to cardiovascular risk (ABSI-z score).

	Very Low	Low	Average	High	Very High	*p*-Value
BMI	24.20 ± 2.89	21.54 ± 2.93	22.77 ± 3.97	21.64 ± 2.14	22.85 ± 3.50	0.320
Relative fat mass	27.22 ± 3.93	18.30 ± 5.13	21.78 ± 7.87	21.47 ± 7.63	18.32 ± 7.60	0.056
Total fat mass	19.36 ± 3.69 ^a^	11.14 ± 3.29 ^b^	14.77 ± 6.48 ^ab^	13.02 ± 4.16 ^b^	12.35 ± 6.16 ^b^	0.012 *
Fat free mass	52.75 ± 13.23	50.94 ± 12.39	52.92 ± 13.87	49.25 ± 10.45	53.58 ± 6.97	0.872
Muscle mass	25.07 ± 7.81	23.68 ± 6.77	25.14 ± 7.78	22.74 ± 6.15	25.40 ± 3.94	0.825
Abdominal girth	82.90 ± 8.03	78.25 ± 12.73	79.37 ± 10.11	75.66 ± 8.68	81.36 ± 8.83	0.357
Weight:	72.11 ± 15.78	62.08 ± 13.18	66.82 ± 17.46	62.28 ± 8.61	65.94 ± 9.66	0.411
Height	171.60 ± 11.42	169.00 ± 6.48	170.25 ± 7.85	169.60 ± 10.70	170.00 ± 7.22	0.986
Visceral fat	1.09 ± 0.62	0.69 ± 0.92	0.77 ± 0.46	0.63 ± 0.47	0.90 ± 0.96	0.550

One-way analysis of variance (ANOVA) was performed, followed by Tukey’s post hoc test for multiple comparisons, and the data are presented as mean ± SD, n = 48 men. The adjusted body composition index (ABSI-z score) was used as a marker of cardiovascular risk, categorizing the participants into five groups: very low, low, average, high, and very high risk. The means in a row without a common superscript letter differ significantly (*p* < 0.05 *).

**Table 5:** Body composition of women according to cardiovascular risk (ABSI-z score).

	Very Low	Low	Average	High	Very High	*p*-Value
BMI	21.92 ± 2.59	21.86 ± 2.77	22.65 ± 3.74	22.51 ± 4.27	22.14 ± 3.77	0.736
Relative fat mass	27.88 ± 5.26	27.22 ± 5.57	27.02 ± 8.14	28.15 ± 8.43	27.03 ± 8.56	0.917
Total fat mass	16.37 ± 5.03	16.36 ± 4.91	16.93 ± 7.64	18.16 ± 8.69	16.69 ± 7.86	0.738
Fat free mass	41.71 ± 4.93	42.85 ± 5.83	44.19 ± 8.18	44.33 ± 7.41	43.32 ± 7.93	0.370
Muscle mass	18.31 ± 2.95	19.08 ± 3.37	19.81 ± 4.86	20.07 ± 4.26	19.47 ± 4.54	0.272
Abdominal girth	70.20 ± 6.94 ^a^	74.70 ± 6.81 ^ab^	78.00 ± 9.06 ^bc^	80.04 ± 10.92 ^cd^	82.85 ± 9.36 ^d^	<0.001 ***
Weight:	58.09 ± 7.69	59.21 ± 8.77	61.12 ± 12.07	62.50 ± 13.01	60.14 ± 12.43	0.372
Height	162.71 ± 4.74	164.44 ± 6.56	163.98 ± 8.26	166.54 ± 6.16	164.51 ± 8.51	0.154
Visceral fat	0.42 ± 0.45 ^a^	0.49 ± 0.49 ^ab^	0.61 ± 0.47 ^ab^	0.82 ± 0.91 ^bc^	0.96 ± 0.71 ^c^	<0.001 ***

One-way analysis of variance (ANOVA) was performed, followed by Tukey’s post hoc test for multiple comparisons, and the data are presented as mean ± SD, n = 248 women. The adjusted body composition index (ABSI-z score) was used as a marker of cardiovascular risk, categorizing the participants into five groups: very low, low, average, high, and very high risk. The means in a row without a common superscript letter differ significantly (*p* < 0.001 ***).

**Table 6:** Total body composition according to cardiovascular risk (ABSI-z score).

	Very Low	Low	Average	High	Very High	*p*-Value
BMI	22.34 ± 2.77	21.83 ± 2.75	22.67 ± 3.74	22.29 ± 3.84	22.26 ± 3.71	0.794
Relative fat mass	27.76 ± 5.02	26.52 ± 5.99	26.36 ± 8.23	26.45 ± 8.68	25.56 ± 8.98	0.645
Total fat mass	16.92 ± 4.92	15.95 ± 4.99	16.66 ± 7.49	16.86 ± 8.08	15.95 ± 7.73	0.891
Fat free mass	43.71 ± 8.21	43.48 ± 6.73	45.28 ± 9.39	45.58 ± 8.47	45.05 ± 8.47	0.577
Muscle mass	19.53 ± 4.92	19.44 ± 3.84	20.48 ± 5.52	20.75 ± 4.89	20.48 ± 4.95	0.496
Abdominal girth	72.50 ± 8.62 ^a^	74.98 ± 7.30 ^ab^	78.17 ± 9.12 ^bc^	78.93 ± 10.50 ^bc^	82.60 ± 9.22 ^c^	<0.001 ***
Weight:	60.64 ± 10.93	59.44 ± 9.04	61.83 ± 12.83	62.44 ± 11.97	61.12 ± 12.14	0.701
Height	164.32 ± 7.21	164.80 ± 6.61	164.76 ± 8.41	167.32 ± 7.59	165.44 ± 8.51	0.253
Visceral fat	0.54 ± 0.54 ^a^	0.51 ± 0.52 ^a^	0.63 ± 0.47 ^ab^	0.78 ± 0.82 ^ab^	0.95 ± 0.75 ^b^	0.001 **

One-way analysis of variance (ANOVA) was performed, followed by Tukey’s post hoc test for multiple comparisons, and the data are presented as mean ± SD, n = 296 total. The adjusted body composition index (ABSI-z score) was used as a marker of cardiovascular risk, categorizing the participants into five groups: very low, low, average, high, and very high risk. The means in a row without a common superscript letter differ significantly (*p* < 0.01 **, *p* < 0.001 ***).

**Table 7:** Description of compliance with the healthy eating criteria among students.

Healthy Eating Criteria	Men (%)	Women (%)	Total (%)	*p*-Value
Fish and shellfish (3–4 servings/week)				
Yes No	15 (31.3%) 33 (68.7%)	61 (24.8%) 187 (75.2%)	76 (25.9%) 220 (74.1%)	0.450
Meats (3–4 servings/week)				
Yes No	35 (72.9%) 13 (27.1%)	196 (80.0%) 52 (20.0%)	231 (78.8%) 65 (21.2%)	0.365
Eggs (3–4 servings/week)				
Yes No	17 (35.4%) 31 (64.6%)	63 (25.6%) 185 (74.4%)	80 (27.2%) 216 (72.8%)	0.223
Legumes (2–4 servings/week)				
Yes No	13 (27.7%) 35 (72.3%)	42 (17.4%) 206 (82.6%)	55 (19.0%) 241 (81.0%)	0.149
Nuts (3–7 servings/week)				
Yes No	20 (41.7%) 28 (58.3%)	70 (28.5%) 178 (71.5%)	90 (30.6%) 206 (69.4%)	0.100
Dairy (2–4 servings/day)				
Yes No	42 (87.5%) 6 (12.5%)	210 (86.8%) 38 (13.2%)	252 (86.9%) 44 (13.1%)	1.000
Vegetables (≥2 servings/day)				
Yes No	22 (45.8%) 26 (54.2%)	114 (47.1%) 134 (52.9%)	136 (46.9%) 160 (53.1%)	0.997
Fruits (≥3 servings/day)				
Yes No	37 (77.1%) 11 (22.9%)	170 (69.1%) 78 (30.9%)	207(70.4%) 89 (29.6%)	0.350
Bread, cereals, rice, pasta, and potatoes (4–6 servings/day)				
Yes No	39 (90.7%) 9 (9.3%)	212 (89.8%) 36 (10.2%)	251 (90.0%) 45 (10.0%)	1.000

## Data Availability

The datasets generated and/or analyzed during the current study are not publicly available owing to the inclusion of confidential data but are available from the corresponding author upon reasonable request.
